# Serum exosomal miR-34a as a potential biomarker for the diagnosis and prognostic of hepatocellular carcinoma

**DOI:** 10.7150/jca.57205

**Published:** 2022-02-21

**Authors:** Shuying Chen, Yinqi Mao, Wei Chen, Chenbin Liu, Han Wu, Jingjun Zhang, Shenghao Wang, Chengpan Wang, Yong Lin, Yuan Lv

**Affiliations:** 1 Huashan Hospital, Fudan University, 12 Wulumuqi Middle Road, Shanghai 200040, People's Republic of China.; 2 School of medicine, Shanghai Jiao Tong University, 227 Chongqing South Road, Shanghai 200025, People's Republic of China.; 3 School of Basic Medical Sciences, Fudan University, 130 Dongan Road, Shanghai 200032, People's Republic of China.

**Keywords:** hepatocellular carcinoma, exosome, miR-34a, molecular biomarker, diagnosis, prognosis

## Abstract

**Background:** Circulating exosomal microRNAs (miRNAs) are considered as potentially non-invasive biomarkers for early detection and prognosis of cancers. Due to the lack of highly sensitive and specific molecular markers, a lot of patients with hepatocellular carcinoma are diagnosed in advanced stages. This study aims to explore the expression mode and clinical detection value of serum exosomal miR-34a in HCC, providing new potential targets and theoretical basis for the early diagnosis and prognosis monitoring of hepatocellular carcinoma.

**Methods:** The expression of serum exosomal miR-34a in 60 HCC patients before and after operation and 60 healthy examiners was abstracted and detected by ultracentrifugation and real-time quantitative PCR. Using ROC analysis, Kaplan-Meier survival analysis and Cox regression analysis, the value of serum exosomal miR-34a on diagnosis and prognosis in HCC patients was assessed.

**Results:** The expression level of serum exosomal miR-34a in preoperative patients was reduced significantly comparing with that in healthy examiners and postoperative patients (P<0.01; P<0.05). Moreover, the decrease of serum exosomal miR-34a was correlated significantly with differentiation degree, TNM stage, tumor infiltration depth and lymph node metastasis(P<0.05), but had no statistical differences with gender, age, ALT, AST, viral infection, cirrhosis and tumor size of HCC patients (P>0.05). At the same time, the combination of serum exosomal miR-34a and α-fetoprotein (AFP) showed high capability on diagnosis to distinguish healthy examiners and HCC patients through ROC analysis. The overall survival of patients with lower expression of serum exosomal miR-34a was worse than that of patients with high level expression by Kaplan-Meier survival analysis (P<0.05). Univariate and multivariate Cox regression analysis both showed that serum exosomal miR-34a was independently related to OS.

**Conclusions:** Collectively, serum exosomal miR-34a is significantly down-regulated in HCC patients and might be a novel noninvasive biomarker for diagnosis and prognosis of HCC.

## Introduction

Hepatocellular carcinoma (HCC) is still one of the tumors with the highest mortality rate. Early diagnosis and prognosis of it can effectively improve the survival rate of patients 1. At present, alpha-fetoprotein test combined with ultrasound examination is mainly way to early diagnosis and prognosis of HCC. But the sensitivity and specificity of alpha-fetoprotein as a HCC marker is low, and ultrasound examination is susceptible to subjective imaging physicians, which leading to the frequent missing or misdiagnosis clinically. These greatly restrict early diagnosis 2. Therefore, there is an urgent need for biomarkers with high sensitivity and high specificity for early diagnosis and prognosis of HCC.

Exosome is a type of small vesicles with a diameter of about 30-150 nm that are secreted by cells. It contains nucleic acids, lipids and proteins and presents in various body fluids such as blood, urine and cerebrospinal fluid 3. It has been reported that exosome produced by tumors carries a large number of molecules that can regulate the physiological activities and participate in physiological changes of tumor cells, such as the invasion, metastasis and drug resistance. Thus it plays a key role especially in the occurrence and development of HCC 4, 5. Therefore, exosome in circulating blood is a valuable non-invasive biomarker.

According to reports, exosomes in circulating blood contain a large number of miRNAs 6. MiRNA is a type of endogenous non-coding small RNA with a size of about 22 nt, which can complementally bind to the 3'end sequence of the target molecule, regulate the expression of the target molecule and participate in a variety of biological processes *in vivo* 7. Studies have shown that exosomal miRNA dysregulation in the tumor environment leads to the changes in the physiological functions of tumor cells. So it is a potential biomarker for tumor diagnosis and prognosis 8-10. As a member of the miRNA family, miR-34a participates in mediating the proliferation, invasion, metastasis and EMT of HCC through regulating many target genes and downstream molecules. It is a potential diagnostic and prognostic biomarkers in the early stage of HCC 11-13. However, there are few reports about the use of miR-34a in serum for the diagnosis and prognosis of HCC, while the reports of miR-34a in serum exosomes for the diagnosis and prognosis of HCC are even rare.

In order to explore the worth of exosomal miR-34a in circulating blood in the early diagnosis and prognostic monitoring of primary hepatocellular carcinoma, we discussed the expression pattern of serum exosomal miR-34a in HCC and its correlation with clinicopathological parameters and evaluated its diagnostic and prognostic value.

## Methods

### Patient and clinical samples

This research was supported by Huashan Hospital affiliated to Fudan University. Serum samples from 60 healthy people and 60 HCC patients from Huashan Hospital were collected preoperative and postoperative. The age and gender of healthy people matched those of patients. All subjects obtained written consent. The research protocol has been approved by the institutional review board of the hospital ethics committee. The clinical characteristics of the subjects are listed in Table 1. And 60 cases of HCC patients were followed up for 6-60 months.

### Isolation of exosomes from serum samples

Peripheral blood was collected and centrifuged at 4,000 rpm for 10 minutes at 4 °C to obtain the upper serum by ultracentrifugation. Then the upper serum was centrifuged at 300 g for 10 minutes, 2000 g for 10 minutes and 10000 g for 10 minutes at 4 °C. The precipitate was discarded and the supernatant was retained. Use an ultrafiltration tube to perform further concentration and centrifugation at 4000 rpm for 20 minutes. Transfer the supernatant to an ultracentrifuge tube, centrifuge at 100000 g for 2 hours, discard the supernatant and resuspend the pellet with 100 ul PBS to obtain serum exosomes.

### Transmission Electron Microscope (TEM)

The exosomal pellet was resuspended in PBS and placed on the copper mesh of the electron microscope. After incubating for 10 minutes at room temperature, use 1% uranyl acetate to negative stain for 10 minutes. Check and take pictures with a transmission electron microscope.

### Western blot analysis

The reagent test kit from abcam was used for precipitation and extraction of exosomes, and the western blot analysis was performed with exosome extract as previously described.

### Serum miRNA quantification

RNA from exosomal pellets was isolated by using isothiocyanate-phenol/chloroform. Real-time quantitative fluorescent RT-PCR (qRT-PCR) was performed using the SYBR Premix Ex Taq (Takara) kit on the ABI 7500 detection system. The cel-miR-39 was taken as the internal reference. The primers of miR-34a and cel-miR-39 were purchased from Guangzhou Ruibo Biotechnology Co., Ltd.

### Statistical Analysis

The *t* test and rank sum test were performed to determine the difference in serum miR-34a levels. ROC working curve analysis, Kaplan-Meier survival analysis and Cox regression analysis were used to evaluate the diagnostic and prognostic value of miR-34a in patients with HCC. All statistical analyses were performed using SPSS 19 and STATA 11.0. P<0.05 was considered as a significant difference. Figures were processed by STATA 11.0 and Photoshop CS.

## Results

### Characterization of isolated serum exosomes

**Figure 1:**
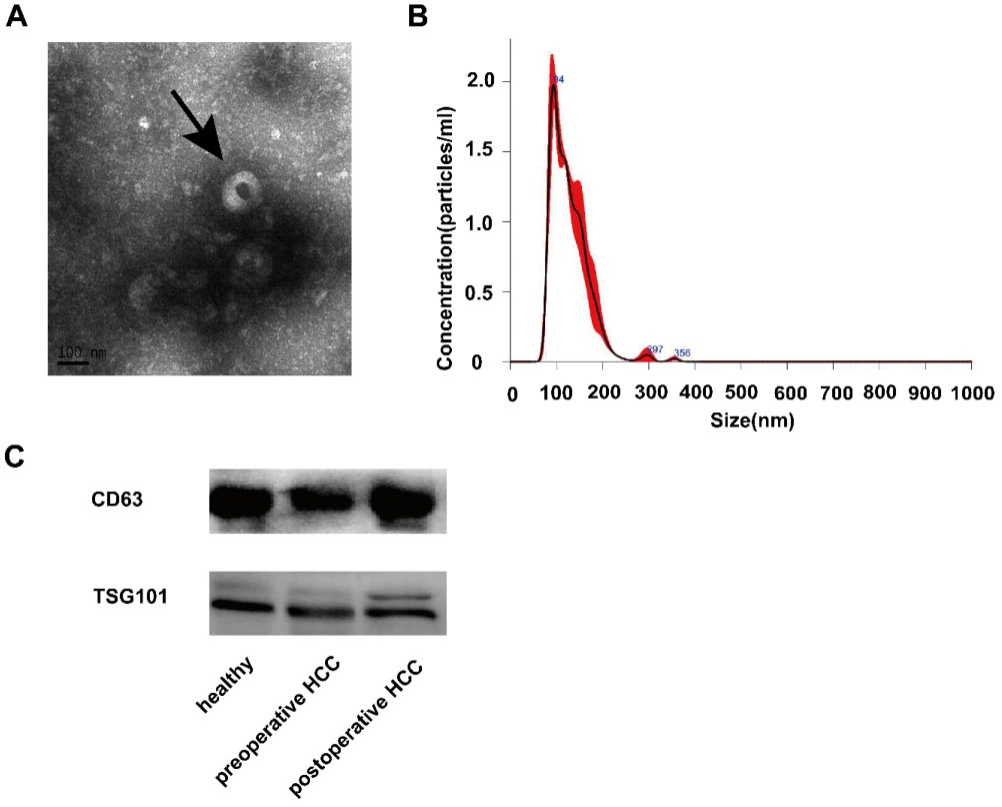
** Expression and identification of serum exosomes. (A)** Morphological characteristics of serum exosomes were observed by transmission electron microscope (The arrow refers to an exosome). **(B)** The size information of serum exosomal particles was measured by Nanosight. **(C)** Identification of serum exosomal marker proteins (CD63 and TSG101) was identificated by western blotting.

To ensure the efficiency and quality of serum exosomes separation, we performed characterization analysis through TEM. Electron microscopy analysis of exosomes isolated from serum samples showed that the size of their circular structures varied from 50 to 150 nm (Fig. 1A), which was consistent with the characteristics of exosomes previously reported by Nanosight (Fig. 1B). In addition, the western blot analysis to detect specific exosomal protein markers CD63 and TSG101 confirmed the existence of exosomal characterization (Fig. 1C). These results confirmed the successful isolation of exosomes from serum samples.

### Serum exosomal miR-34a expression in preoperative patients with HCC is significantly increased

**Figure 2:**
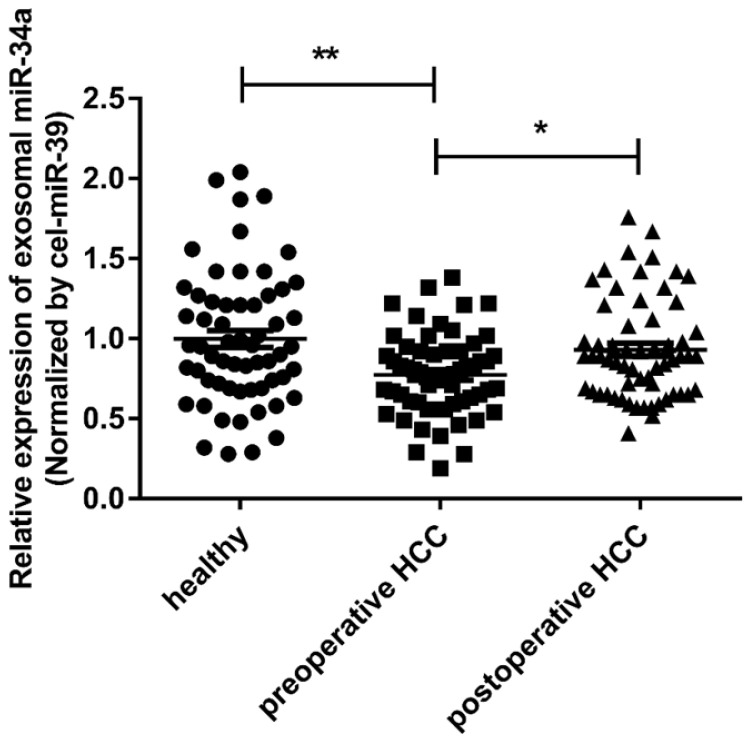
The expression of exosomal miR-34a in serum was measured by qRT-PCR. *P<0.05, **P<0.01, compared with the negative control.

In order to determine whether the miR-34a in the serum was derived from exosomes or serum, RNA was extracted from the exosomal precipitates and exosomal-free supernatants isolated from 60 healthy human serum samples. The expression of miR-34a was checked by qRT-PCR. The concentration of miR-34a in exosomes was significantly higher than that in the supernatant without exosomes (P <0.05, Fig. 2). In order to check the expression of miR-34a in whole serum, RNA was extracted from 10 serum samples that used above, and the expression of miR-21 was quantified by qRT-PCR. The concentration of miR-34a in whole serum samples was lower than exosomes, but higher than the supernatant without exosomes (Fig. 2).

To discuss whether the exosomal miR-34a has the potential to become a biomarker, the expression levels of miR-34a in serum exosomes of 60 HCC patients' preoperative and postoperative and 60 healthy people were analyzed. The results of qRT-PCR showed that the preoperative serum exosomal miR-34a expression level was significantly lower than that of healthy subjects and postoperative HCC patients (P<0.01; P<0.05) (Fig. 2). This result suggests that the exosomal miR-34a in the peripheral blood may become a serum biomarker for early screening or early diagnosis of HCC.

### Serum exosomal miR-34a is related to the clinicopathological characteristics of HCC and is less affected by changes in the patient's physiological state and other liver diseases

A Spearman correlation analysis was performed to determine the relative expression of serum exosomes miR-34a to better understand the potential role of serum exosomes miR-34a in the occurrence and development of HCC and the extent which affected by changes in physiological conditions. The digital 0.77 is the cut-off value. 30 cases of HCC patients with low expression of miR-34a in serum and 30 cases with high expression of miR-34a proved that the potential between serum exosomal miR-34a levels and various clinicopathological characteristics of HCC was analyzed. The result showed that the down-regulation of serum exosomal miR-34a was significantly related to the degree of differentiation, TNM stage, tumor invasion depth, lymph node metastasis and vascular invasion (P<0.05), while there was no statistical difference in patient gender, age, ALT, AST, viral infection, cirrhosis and tumor size (P>0.05) (Table 1).

### Serum exosomes miR-34a has early diagnostic value

**Figure 3:**
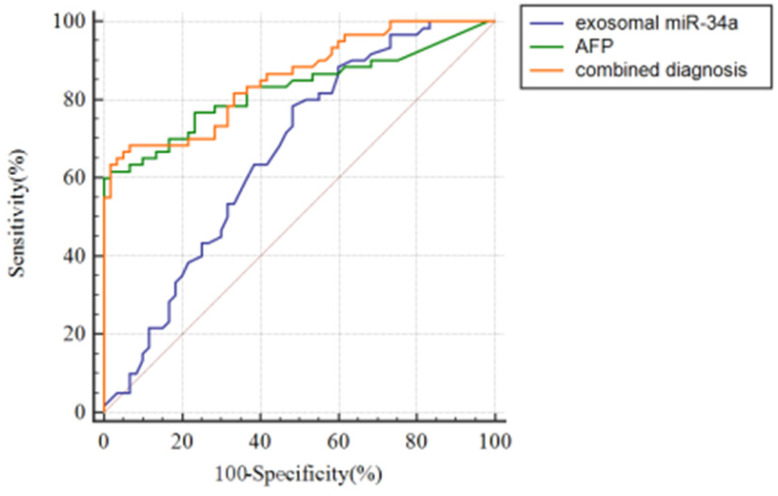
ROC curve analysis of serum exosomes miR-34a, AFP and the combined application of the two to diagnose HCC.

To discuss the potential value of serum exosomal miR-34a for the early diagnosis of HCC, receiver operating curve (ROC) was used to analyze the serum exosomal miR-34a expression level of healthy people and preoperative HCC patients. Fig. 3 and Table 2 show the ROC curve for the diagnosis of HCC. The results showed that the AUC values of serum exosomes miR-34a, AFP and their combined application were 0.664±0.0499 (P<0.01), 0.826±0.0396 (P<0.001) and 0.855±0.0337 (P<0.001) (Table 2). The sensitivity and specificity of serum exosomes miR-34a, AFP and their combined detection for the diagnosis of HCC were 78.3% and 51.7%, 61.7% and 98.3%, 68.33% and 93.33%, respectively (Table 2). In terms of AUC value, serum exosomal miR-34a was statistically different from the other two groups (P<0.05/P<0.001), but there was no statistical difference between the AFP group and the combination group (Table 3). However, the AUC value in the combination group was higher than that in the exosomal miR-34a or AFP group alone and the sensitivity and specificity in the combination group were higher than those in the AFP or exosomal miR-34a alone, which reminded us the combination of serum exosomes miR-34a and AFP can help improve the AUC value, specificity and sensitivity of the diagnosis of HCC patients. It has potential in early clinical diagnosis and can distinguish HCC patients from health groups (Table 2 and Fig. 3).

### Serum exosomes miR-34a correlates with patient prognosis

**Figure 4:**
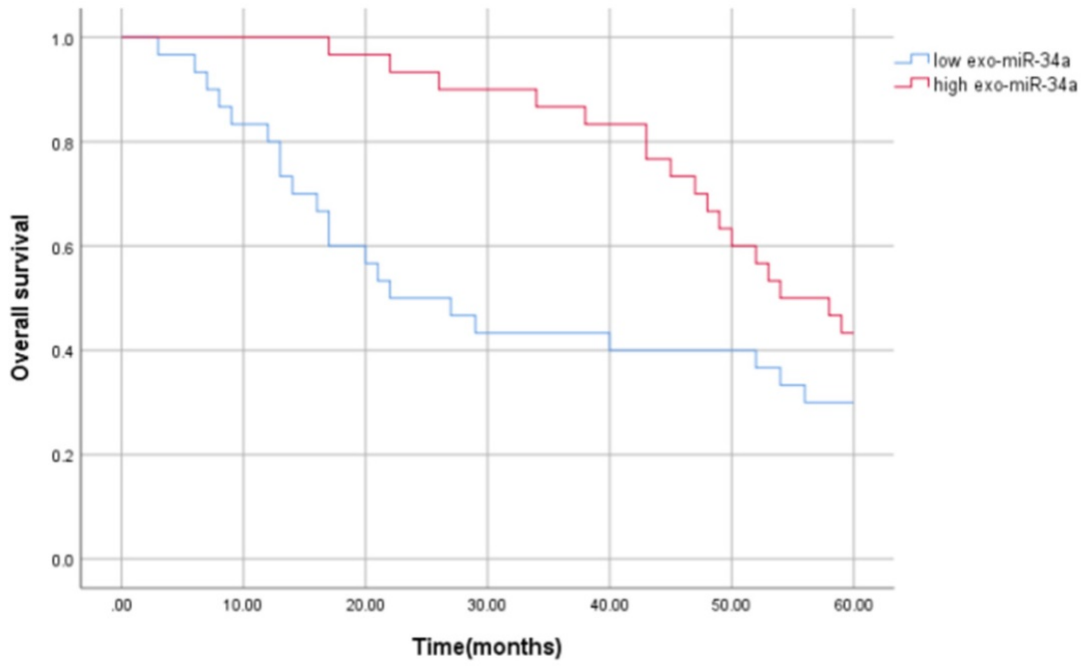
Kaplan-Meier curve of the relationship between serum exosomal miR-34a expression and disease-free survival of patients with HCC.

In order to evaluate the prognostic value of serum exosomal miR-34a in HCC patients, Kaplan-Meier survival analysis and univariate and multivariate Cox regression analysis were used to prove the potential relationship between the serum exosomal miR-34a expression and its prognosis. The results showed that compared with the high expression group of serum exosomes miR-34a, patients with low expression of serum exosomes miR-34a had worse overall survival (OS) (P<0.05), and serum exosomal miR-34a level is independently related to OS. The average survival time of patients with low serum exosomal miR-34a expression was (33.200±3.991) months, the median survival time was 22.000 months, and the 5-year survival rate was 30%, while the average survival time of patients with high serum exosomal miR-34a expression (50.600±2.223) months, the median survival time was 54.00 months, and the 5-year survival rate was 43.3%. The survival rate and survival time of patients with low expression of serum exosomes miR-34a were lower than those with high expression, and the difference was statistically significant as showed in Figure 4 (P = 0.042). Univariate Cox regression analysis showed that serum exosomal miR-34a level was closely related to the OS (P=0.042). Further multivariate analysis showed that serum exosomal miR-34a expression (P = 0.047) can be used as an independent factor for predicting the poor prognosis of HCC patients. The relative risk HR=1.918, indicating that patients with low serum exosomal miR-34a expression are 1.918 times as likely to die as patients with high expression, and the 95% confidence interval (95% CI) of HR is 1.009~ 3.646 (Table 4). These results indicate that serum exosomal miR-34a is an independent factor affecting the prognosis of patients with liver cancer (Table 4).

## Discussion

Hepatocellular carcinoma is still one of the tumors with the highest mortality rate. Early diagnosis and prognosis can effectively improve the survival rate of patients 1. At present, the early diagnosis and prognosis of HCC mainly rely on alpha-fetoprotein combined with ultrasound examination in clinical practice. But the sensitivity and specificity of alpha-fetoprotein as a liver cancer marker is low and early ultrasound examination is difficult and inaccurate for the diagnosis of small lesions, and it is easily affected by the subjective judgment of imaging physicians, which leading to frequent missed or misdiagnosed clinically. These greatly restrict early diagnosis. Therefore, there is an urgent need for biomarkers with high sensitivity and high specificity for early diagnosis and prognosis monitoring of HCC.

At present, a large number of studies have shown that a variety of miRNA disorders are involved in the occurrence and development of cancer. These disordered miRNAs mediate and participate in the physiological functions of tumor cells. For example, miR-222 increases the invasion of ovarian cancer cells by acting on the target gene PTEN 14. MiR-214 regulates the proliferation and metastasis of thyroid tumor cells by acting on WWOX and PTEN 15. MiR-543 accelerates the proliferation of nasopharyngeal carcinoma cells by targeting JAM-A 16. Cancer cells can secrete exosomes, which are rich in miRNAs, and abnormal expression of exosomal miRNAs can be detected in various body fluids such as serum. For example, serum exosomes miR-196a and miR-1246 are upregulated in pancreatic cancer 17. Serum exosomes miR-25-3p and miR-92a-3p are up-regulated in HCC 18. These provide a new opportunity to find cancer biomarkers.

Serum miR-34a is a more sensitive and specific indicator for diagnosing primary hepatocellular carcinoma 19, as well as a protective factor in the development of liver cancer 12, 13. It can be used as a prognostic indicator. Therefore, we took serum exosomes miR-34a as the research object, studied the expression of miRNA-34a in serum exosomes of HCC patients and healthy volunteers, and further evaluated the diagnostic and prognostic value of miR-34a in HCC patients. We observed that the expression of miR-34a in exosomes of preoperative HCC patients was significantly lower than that of healthy people and postoperative patients. In addition, this study showed that the down-regulation of serum exosomal miR-34a was significantly related to the degree of differentiation, TNM stage, tumor invasion depth, lymph node metastasis and vascular invasion and there was no obvious relationship to patient gender, age, ALT, AST, viral infection, liver cirrhosis, and tumor size, etc. Further statistical analysis showed that the combination of serum exosomal miR-34a and AFP can help improve the AUC value, specificity and sensitivity of the diagnosis of HCC, and serum exosomal miR-34a is an independent factor affecting the prognosis of HCC patients. The above results indicate that serum exosomal miR-34a has potential value in early clinical diagnosis and prognosis, which are consistent with the results published by Bharali D et al. 19. However, the number of cases involved in this study is limited, and there are certain limitations in sample selection, which needs to be increased for further verification.

The main way that miRNA produce biological effects in the body is to combine with its downstream confirmed target molecules to affect the physiopathological process of the disease. Regarding the report on the downstream related target genes of miR-34a and their signaling pathways, a luciferase reporter gene, RT-qPCR and western blotting experiments confirmed that miR-34a can target the HK1 molecule and reduce the expression level of HK1, promoting the vitality and proliferation of hepatocellular carcinoma cells^20^. The other study is to evaluate the functional roles of miR-34a by applying microarray profiling and analyzing miR-34a's predicted targets and related biological pathways in HCC 21. The researchers explored 1,000 miR-34a-related target genes and five important signal pathways, of which CCND1 and Bcl-2 act as essential central genes. In the integration analysis, it is found 61 central genes and 5 important pathways, including cell cycle, cytokine-cytokine receptor interaction, notching pathway, p53 pathway and focal adhesion. These target genes and signal pathways indicate potentially related functions of miR-34a in HCC. This part of the experiment mainly explores the correlation between the expression of exosomal miR-34a and the clinicopathological parameters of HCC patients from the clinical level. In the follow-up, we will further explore the functions of miR-34a from the molecular mechanism level in combination with the cellular and animal levels.

In summary, the results show that the expression of miR-34a in exosomes is down-regulated in preoperative HCC patients compared to postoperative patients and healthy people. Thus it can be used as a non-invasive biomarker with great potential for screening and monitoring HCC.

## Conclusions

In summary, the results show that the expression of miR-34a in exosomes is down-regulated in preoperative HCC patients compared to postoperative patients and healthy people. Thus it can be used as a non-invasive biomarker with great potential for screening and monitoring HCC.

## Figures and Tables

**Table 1:** Correlation between expression levels of exosomal miR-34a in preoperative serum and clinicopathological factors of patients with hepatocellular carcinoma. *P*<0.05 indicates significance

Clinicopathologic Parameters	Low Expression of exosomal miR-34a (n=30)	High Expression of exosomal miR-34a (n=30)	*P* value
**Gender**			
Male	18	16	0.795
Female	12	14	
**Age (year)**			
≤55	13	11	0.792
>55	17	19	
**AFP (ng/ml)**			
≤20	10	13	0.596
>20	20	17	
**Viral infection**			
with	20	12	0.069
without	10	18	
**ALT (U/L)**			
≤40 U/L	11	19	0.120
>40 U/L	19	11	
**AST (U/L)**			
≤40 U/L	12	19	0.120
>40 U/L	18	11	
**Cirrhosis**			
with	20	14	0.192
without	10	16	
**Tumor size (cm)**			
≤5	9	16	0.115
>5	21	14	
**TNM stage**			
I-II	10	19	0.038*
III-IV	20	11	
**Differentiation**			
middle or high	7	17	0.017*
low	23	13	
**Lymph node metastasis**			
positive	21	11	0.019*
negative	9	19	
**Vascular invasion**			
positive	22	12	0.018*
negative	8	18	

**Table 2:** The AUC value of exosomal miR-34a, AFP and the combination for hepatocellular carcinoma. ^**^P<0.01; ^***^P<0.001

Index	AUC ( 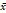 ± s)	*P* value	95% CI	Sensitivity (%)	Specificity (%)
exo-miR-34a	0.664±0.0499	0.0010**	0.572~0.747	78.3	51.7
AFP	0.826±0.0396	<0.0001***	0.746~0.889	61.7	98.3
exo-miR-34a + AFP	0.855±0.0337	<0.0001***	0.780~0.913	68.33	93.33

**Table 3:** Pairwise analysis of the AUC values of different groups for diagnosis of hepatocellular carcinoma. ^*^P<0.05; ^***^P<0.001

Index	difference	Standard deviation	Z value	*P* value
exo-miR-34a: AFP	0.162	0.0631	2.570	0.0102*
exo-miR-34a: (exo-miR-34a + AFP)	0.192	0.0464	4.134	<0.0001***
AFP: (exo-miR-34a + AFP)	0.0296	0.0238	1.243	0.2140

**Table 4:** Univariate and multivariate Cox regression analysis of factors affecting the overall survival of 60 HCC patients

Clinicopathological parameters	Univariate			Multivariate		
	*HR* value	95%CI	*P* value	*HR* value	95%CI	*P* value
Exo-miR-34a	0.521	0.274~0.992	0.047	1.918	1.009~3.646	0.047
Gender	1.263	0.659~2.423	0.481			
Age	1.247	0.644~2.413	0.512			
AFP	1.526	0.770~3.026	0.226			
Viral infection	1.242	0.655~2.354	0.507			
ALT	1.384	0.729~2.624	0.320			
AST	0.971	0.513~1.836	0.927			
Cirrhosis	0.977	0.515~1.851	0.942			
Tumor size	1.435	0.742~2.777	0.283			
TNM stage	1.735	0.909~3.314	0.095			
Differentiation	1.764	0.900~3.457	0.098			
Lymph node metastasis	1.767	0.920~3.396	0.087			
Vascular invasion	1.002	0.528~1.900	0.995			

## Abbreviations

miRNAs: microRNAs
HCC: hepatocellular carcinoma
ROC: receiver operating curve
qRT-PCR: quantitative reverse-transcription polymerase chain reaction
AFP: alpha fetal protein
